# *Coriandrum sativum* attenuates microglia mediated neuroinflammation and MPTP-induced behavioral and oxidative changes in Parkinson’s disease mouse model

**DOI:** 10.17179/excli2021-3668

**Published:** 2021-04-28

**Authors:** Sushruta Koppula, Ramesh Alluri, Spandana Rajendra Kopalli

**Affiliations:** 1 College of Biomedical and Health Science, Konkuk University, Chungju-Si, Chungcheongbuk Do, 380-701, Republic of Korea; 2 Department of Pharmacy, Vishnu Institute of Pharmaceutical Education and Research, Hyderabad, Telangana, India; 3 Department of Bioscience and Biotechnology, Sejong University, Gwangjin-gu, Seoul 05006, Republic of Korea

**Keywords:** Coriandrum sativum, microglia, lipopolysaccharide, interleukin, oxidative stress, MPTP, Parkinson's disease

## Abstract

*Coriandrum sativum* Linn. (family: Umbelliferae; *C. sativum)*, is a potential herb widely used as a spice and traditional medicine. In the present work, the effects of *C. sativum* fruit extract (CSE), against lipopolysaccharide (LPS)-stimulated BV-2 microglia-mediated neuroinflammation *in vitro *and 1-methyl-4 phenyl-1, 2, 3, 6-tetrahydropyridine (MPTP)-induced Parkinson's disease (PD) animal model *in vivo *was investigated. LPS-stimulated increase in nitric oxide (NO), inducible NO synthase, cyclooxygenase-2, interleukin-6 and tumor necrosis factor-alpha were significantly (p < 0.05 ~ p < 0.001) inhibited by CSE (25, 50 and 100 μg/mL) in BV-2 microglial cells. Further, CSE inhibited the LPS-induced nuclear factor of kappa-beta activation and IκB-α phosphorylation in BV-2 microglia. *In vivo * studies, CSE (100, 200 and 300 mg/kg) ameliorated the MPTP (25 mg/kg, i.p.)-induced changes in locomotor, cognitive and behavior functions evaluated by rotarod, passive avoidance and open field test significantly (p < 0.05 ~ p < 0.001). The MPTP-induced changes in brain oxidative enzyme levels such as superoxide dismutase, catalase, and lipid peroxidation were significantly (p < 0.01 and p < 0.001 at 200 and 300 mg/kg, respectively) restored with CSE treatment. High-performance thin-layer chromatography fingerprinting analysis of CSE exhibited several distinctive peaks with quercetin and kaempferol-3O-glucoside as identifiable compounds. In conclusion, our study indicated that CSE attenuated neuroinflammatory processes in LPS-stimulated microglia *in vitro* and restored the MPTP-induced behavioral deficits and brain oxidative enzyme status *in vivo *proving its therapeutic potential in the treatment of neuroinflammatory and oxidative stress-mediated neurodegeneration seen in PD.

## Introduction

Parkinson's disease (PD) is a progressive neurodegenerative disease characterized by gradual loss of dopaminergic neurons in the substantia nigra region of the brain (de Lau and Breteler 2006[11]; Herrera et al., 2017[23]). Although the underlying mechanisms remain unclear and not well understood, microglial activation, neuroinflammatory responsive molecules released by the brain-resident immune cells in the central nervous system, oxidative stress and reduced antioxidant defense are the most important processes involved in the pathogenesis of PD (Im et al., 2006[25]; Troncoso-Escudero et al., 2018[66]). Currently, there is no effective cure for PD and much of the available therapeutic agents offer only symptomatic relief and failed to restore or halt the disease progression, rather produced various unwanted side-effects upon long-term usage (Samii et al., 2004[59]). Therefore, alternative therapy using natural herbs with immense traditional medicinal values might be necessary for the prevention and treatment of PD (Javed et al., 2019[30]). Evidence suggests a number of potential natural herbs including dietary components, functional foods were known to improve brain function by modulating the neuroinflammatory and neurodegenerative signaling processes (Gómez-Pinilla, 2008[19]; Angeloni and Vauzour, 2019[4]; Fei et al., 2020[16]; Mohd Sairazi and Sirajudeen, 2020[45]). 

*Coriandrum sativum* Linn (*C. sativum*) from the family Umbelliferae/Apiaceae commonly known as “Coriander” is a traditional medicinal herb and one of the World's oldest known spices (French, 1971[17]; Mathias, 1994[40]). The fruits of *C. sativum* are known to be an important source for thousands of years with immense medicinal benefits including carminative, halitosis, diuretic, antibilious, antiulcer and antirheumatism effects (Hegi, 1926[22]). Besides the use of *C. sativum* as a flavor and spice in culinary practices, it is also used in folk medicine to lower cholesterol, blood sugar, blood pressure, diarrhea, mouth ulcers, anemia, menstrual and digestive problems in Asian countries for thousands of years (Cicin, 1962[9]; Al-Snafi, 2016[3]). Pharmacologically, *C. sativum* has been reported to possess antioxidant (Deepa and Anuradha, 2011[12]), anticancer (Chithra and Leelamma, 2000[8]), antimicrobial (Matasyoh et al., 2009[39]), antidiabetic (Sushruta et al., 2007[61]), hepatoprotective (Pandey et al., 2011[49]), diuretic (Aissaoui et al., 2008[1]), antiinflammatory (Wu et al., 2010[70]), anxiolytic (Emamghoreishi et al., 2005[15]; Sahoo and Brijesh, 2020[58]), antihypertensive (Jabeen et al., 2009[26]), antinociceptive, antiedema (Begnami et al., 2018[6]), memory enhancing (Vasudevan Mani, 2009[67]; Mani et al., 2011[38]), hypolipidemic (Chithra and Leelamma, 2000[8]), and heavy metal detoxification effects (Tellez-Lopez et al., 2017[63]).

With respect to its neurological effects, *C. sativum* improved the learning capacity in new born mice (Zargar-Nattaj et al., 2011[74]), exhibited neuroprotective effects against ischemic-reperfusion insult in rats (Vekaria et al., 2012[68]), enhanced memory in diazepam-, scopolamine-, and aging-induced amnesia in young and aged rats (Vasudevan Mani, 2009[67]; Jasira et al., 2017[29]), ameliorated aging-induced memory impairment in senescence-accelerated mouse-prone 8 mice (Mima et al., 2020[43]), exhibited neuroprotective effects against ischemic-reperfusion injury in rat brain (Vekaria et al., 2012[68]), prevented neuronal damage in pentylentetrazole-induced seizure in rats (Pourzaki et al., 2017[53]), attenuated tacrine-induced orofacial dyskinesia in rats proving its ability in suppressing parkinsonian symptoms (Mohan et al., 2015[44]), and promoted neuroprotection on the rat progeny of mothers exposed to methylmercury (Rodrigues et al., 2019[56]). Moreover, our earlier study indicated that *C. sativum* fruit extract exhibited anti-amnesic effects in scopolamine-induced experimental rats (Koppula and Choi, 2012[33]). 

Despite the pharmacological benefits of *C. sativum *in improving neurological functions, the effect of *C. sativum* against neuroinflammatory responses in stimulated microglia, and neurotoxin-induced behavioral and oxidative imbalance in experimental model of Parkinson's Disease (PD) was not studied. In the present study, the protective effect of *C. sativum *fruit extract against lipopolysaccharide (LPS)-induced BV-2 microglial cells *in vitro* and 1-methyl-4 phenyl-1, 2, 3, 6-tetrahydropyridine (MPTP)-induced oxidative damage and behavioral impairments in mouse model of PD *in vivo* was investigated. 

## Materials and Methods

### Chemicals

Lipopolysaccharide (LPS), dimethyl sulfoxide (DMSO) and 3-(4, 5- dimethylthiazol-2-yl)-2,5-diphenyl-tetrazolium bromide (MTT) and thiobarbituric acid (TBA) were purchased from Sigma-Aldrich, St Louis, MO, USA. DMEM was purchased from Invitrogen, Carlsbad, CA, USA, fetal bovine serum (FBS) from Hyclone, Logan, UT, USA. Specific antibodies against inducible nitric oxide synthase (iNOS), cyclooxygenase (COX)-2, nuclear factor-kappa B (NF-κB), IκB-α, and phosphor (p) -IκB-α were obtained from Bio-Rad, Herculus, CA, USA. 

### Preparation of extract and high performance thin layer chromatography (HPTLC) 

The dried fruit material of *C. sativum* was obtained from M/s Laila Impex, Vijayawada, India and the authenticity of the material was confirmed by taxonomist Chemiloids R & D center, India and stored in our department herbarium (specimen no: CSE-2018). The protocol for extraction was followed as described in our previous report (Koppula and Choi, 2012[33]). The final yield of *C. sativum* fruit extract, hereby named as CSE was 11.06 % and was freely soluble in distilled water for further use in experiments. For HPTLC finger print analysis, CSE 200 mg was weighed and transferred in to 10 mL volumetric flask and made up the mark with methanol, sonicated to dissolve it in methanol. The solution was filtered through a 0.4 µ membrane filter into auto sampler vial. A Camag automatic sampler (ATS 4) with Camag TLC scanner 4 HPTLC system with WinCATS software were used. For identification, the mobile phase was transferred into 10 x 10 cm stationary phase (Silica Gel 60F254) TLC chamber and allowed it to equilibrate for 30 min at room temperature. The sample (15 µL) was applied on to the TLC plate and developed the plate in the TLC chamber in the mobile phase [Ethyl acetate: Methanol: Water (8.2:1:0.8)] about 8 cm from the point of application. After development the plate was dried to remove solvent traces and evaluated the TLC plate at 254 nM.

### Cell culture and cell viability assessment

BV-2 microglia cells were cultured at 37 °C in 5 % CO_2_ in DMEM supplemented with 5 % FBS and antibiotics (Invitrogen). Cells were seeded at a density of 5 ×10^4^ cells/mL and pretreated with CSE at various concentrations ranging from 10-100 μg/mL for 1 h before the addition of LPS (1 μg/mL) in serum free DMEM. An equal volume of sterile water was added to all control treatments. For cell viability, MTT assay was used as described previously (Kim et al., 2011[32]). Briefly, various concentrations of CSE (10, 25, 50, 100 and 200 μg/mL) were incubated for 24 h followed by incubation in dark at 37 °C with MTT for additional 2 h. The medium was carefully removed from the wells and the blue formazan product was dissolved in DMSO. Each experiment was conducted three times. The absorbance was analyzed at 570 nm using a microplate reader (Tecan Trading AG, Switzerland). Using the formula (O.D. of extract treated sample/O.D. of non-treated sample) x 100 %, the cell viability percentage was calculated. 

### Nitric oxide (NO) assay

Production of NO was assayed by measuring the levels of nitrite in the culture supernatant using colorimetric assay with Griess reagent (Green et al., 1982[21]). Briefly, BV-2 cells (2 x 10^5^ cells/mL) were seeded in 500 μL complete culture medium in 6-well plates and treated with the CSE (25, 50 and 100 μg/mL) for 1 h prior to stimulation with LPS (1 μg/mL) for 2 h. The supernatant was removed (50 μL) and reacted with an equal volume of Griess reagent (0.1 % naphthylethylenediamine and 1 % sulfanilamide in 5 % H_3_PO_4_) in 96-well plates at room temperature in the dark. The standard solutions of sodium nitrite prepared in the culture medium was used to determine nitrite concentrations. The absorbance (540 nm) was read using a PowerWavex Microplate Scanning spectrophotometer (Bio-Tek Instrument, Winooski, VT, USA).

### Interleukin (IL)-6 and tumor necrosis factor-alpha (TNF-α) production

CSE (25, 50 and 100 μg/mL) was treated for 1 h to BV-2 microglia cells (1 x 10^5^ cells/well) cultured on 96 well plates and stimulated with LPS (1 μg/mL) for 4 h. The supernatants removed were evaluated for TNF-α and IL-6 levels using TNF-α and IL-6 ELISA kits according to the manufacturer's instructions (BD Biosciences, San Jose, CA, USA).

### Western blotting

Cultured BV-2 cells were washed three times in cold PBS and lysed in a buffer containing 50 mM Tris-HCl, pH 7.4, 1 % (v/v) NP-40, 0.25 % sodium deoxycholate, 150 mM NaCl, 1 mM EDTA, 25 mM NaF, 2 mM Na3VO4 and protease inhibitor cocktail (Complete MiniTM, Roche, Mannheim, Germany) at 4 °C. The lysate was clarified by centrifugation at 10,000 g for 20 min at 4 °C to remove insoluble components. Cell lysates were normalized for protein content using BCA reagent (Pierce, Rockford, IL, USA). Equal amounts of protein were loaded onto 10 % PAGE gels and separated by standard SDS-PAGE procedure. Proteins were transferred to an NC membrane (S&S, Dassel, Germany) and blocked with 5 % non-fat dry milk in TBS. To detect protein expression, the blots were probed with the specific antibodies against iNOS, COX-2, NF-κB, IκB-α, and phosphor (p)-IκB-α followed by the secondary antibodies coupled to horseradish peroxidase (Bio-Rad, Herculus, CA, USA). The detection of β-actin with a specific antibody was used for an internal control. The blots were detected by chemiluminescence using the West-Save substrate (Lab-Frontier, Seoul, Korea) on X-ray film. 

### Animals 

Male Swiss albino mice weighing between 20-25 g were obtained from Vishnu Institute of Pharmaceutical Education and Research, Hyderabad, India and housed in an air conditioned animal room at 23 ± 2 °C with 12/12 h light/dark photoperiod, and given free access to food and water. The animals were kept for seven days in laboratory for habituation. All animal experiments were performed in accordance to the internationally accepted ethical guidelines (Derrell, 1996[13]), and approval from Institutional Animal Ethics Committee (Regd.No: 1358/ERe/S/10/CPCSEA), Hyderabad, India.

### Experimental design

Animals were divided into six groups (n = 10), i.e. vehicle, MPTP (25 mg/kg), CSE (300 mg/kg), MPTP plus CSE 100 mg/kg, MPTP plus CSE 200 mg/kg and MPTP plus CSE 300 mg/kg. MPTP 25 mg/kg (*i.p.*) along with probenecid 250 mg/kg (*i.p.*) was administered for five consecutive days (day 1 - day 5) to induce chronic parkinsonian symptoms in mice as described previously (Jackson-Lewis and Przedborski, 2007[27]). Different doses of CSE (100, 200 and 300 mg/kg) were prepared freshly by dissolving in distilled water and administered on day 1 (1 h prior to MPTP administration) and was continued up to 21 days through oral gavage (*p.o.*). Locomotor behavioral paradigm was evaluated during the course of MPTP administration (day 4) and also at the last phase of the study (day 20). Cognitive paradigm using open field test was evaluated on day 5 and on day 21. Passive avoidance test was evaluated on day 20 (acquisition) and day 21 (retention). After the behavioral experiments, mice were sacrificed by cardiac perfusion and brain tissues were isolated as described previously (Patil et al., 2014[51]). Briefly, the whole brain tissues were rinsed and homogenized with ice-cold phosphate buffer saline (pH 7.4), and centrifuged at -4 °C for 15 min. Aliquots of homogenates were used for estimation of superoxide dismutase (SOD), catalase (CAT) and lipid peroxides (LPO) levels. 

### Rota rod test

For assessing the locomotor coordination in mice, rotarod test was performed (ROTA-ROD for mice 7650 by UGO Basile, Varese, Italy) as described earlier with slight modification (Rozas et al., 1997[57]). In this test, the duration of time that mice maintain their balance on a rotating rod (diameter, 3 cm) was measured. The mice were placed on a horizontal plastic rod and the average time (s) in maintaining their balance on the rotating rod at a speed of 30 rpm for a maximum of 90 s was measured. The rotarod test was performed on days 4 and 20, and the mean duration for each mouse was determined and used for comparison.

### Passive avoidance test

The effects of CSE extract on learning and memory was measured by passive avoidance test apparatus (GEMINI, Model PACS-30, San Diego instruments Int., USA) with slight modifications described previously (Shen et al., 1990[60]). Briefly, animals were acclimatized in the passive avoidance test apparatus for 2-3 min. Acquisition trial was performed by placing the mouse in the illuminated chamber for 30 s followed by computer controlled opening of guillotine door. Upon mice entering into the dark chamber the door was automatically shut and electric foot shock (1 mA) through the grid floor was given for 3 s. After 24 h of the acquisition trial, mice were placed in the light chamber, and the latency time (s) to enter the dark chamber was measured for 600 s (retention trial). Animals retaining in the light chamber throughout the duration were assigned a latency time value of 600 s. 

### Open-field test

Open-field test was performed as described previously (Gould et al., 2009[20]). Briefly, the open field test apparatus (Coulbourn Instruments L.L.C., Holliston, MA, USA) consisted of a wooden box with square arena of 60 × 60 × 60 cm sub-divided into 16 squares, four in the center and 12 along the walls. The test was initiated by placing the mouse at the center of the arena and the number of line crossings, rearing, grooming and duration of immobility was observed for 5 min and cleaning with 70 % ethanol using cotton pads after each test.

### SOD assay

SOD activity assay was performed by pyrogallol autoxidation method as described previously (Li, 2012[36]). Briefly, the reaction mixture 3 mL contained potassium phosphate buffer (2.8 mL; 0.1 M, pH 7.4), tissue homogenate (0.1 mL) and pyrogallol solution (0.1 ml; 2.6 mM in 10 mM HCl). Absorbance was measured at 325 nm for 5 min with 30 s interval. One unit of SOD is described as the amount of enzyme required to cause 50 % inhibition of pyrogallol autoxidation per 3 mL of the assay mixture.

### CAT assay

CAT activity was assessed by the method described previously (Patil et al., 2014[51]). Reaction mixture consisted of 2.0 mL of diluted homogenate in 0.1 M phosphate buffer and 1 mL of 200 mM hydrogen peroxide. The rate of decrease in the absorbance was recorded against the blank at 240 nm for 3 min at 15 s intervals. The results were expressed as U/mg of protein.

### LPO assay 

LPO levels were evaluated by the spectrophotometric method as reported previously (Ohkawa et al., 1979[47]). Tissue homogenates (100 μL) and TCA solution (10 %, 1.5 mL) were mixed for 10 min in the test tubes and centrifuged for 15 min at 5000 rpm. The supernatant was removed and mixed with 1.5 mL of 0.8 % aqueous solution of TBA. The mixture containing tubes was then boiled for 30 min in a water bath and cooled. Absorbance was measured at 532 nm and the LPO levels were expressed in terms of nmol/mg of protein.

### Statistical analysis

Data are presented as mean ± standard error of the mean (S.E.M). Results were calculated using one-way analysis of variance (ANOVA) followed by the Dunnett's multiple range tests (GraphPad Software Inc., San Diego, CA, USA). Values of p < 0.05 was considered statistically significant.

## Results

### Effect of CSE on cell viability and LPS-induced NO release in BV-2 microglial cells

**Figure 1:**
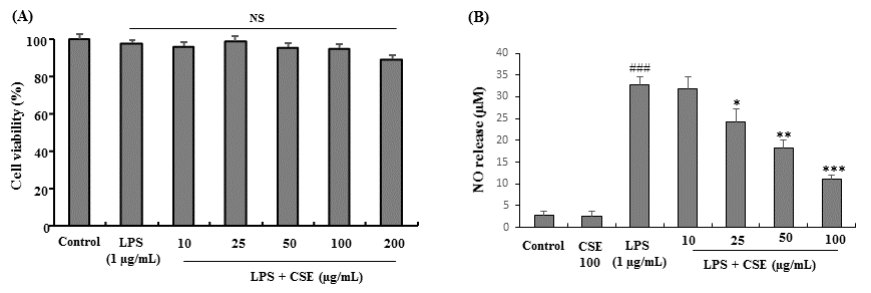
Effect of CSE on the cell viability and NO production in LPS-stimulated BV-2 cells. Cells were incubated with indicated concentrations of CSE for 1 h before incubation with LPS (1 µg/mL) for 24 h. Cell viability was evaluated using the MTT assay. Results are shown as percentage of control samples (A). The nitrite levels were measured as NO release (µM) in the culture media using the Griess reaction (B). Data are presented as the mean ± S.E.M. (n =6). ^###^*P*<0.001, vs. control group and ^*^p < 0.05, ^**^p < 0.01 and ^***^p < 0.001 vs. LPS treated group. NS: Not significant; CSE: *Coriandrum sativum *extract

CSE treated at various concentrations (10, 25, 50, 100 and 200 µg/mL) with LPS (1 µg/mL) and LPS alone treated cells did not show any signs of cytotoxicity or influenced the overall cell viability on BV-2 cells as determined by MTT assay. Although not significant CSE at 200 µg/mL showed mild cytotoxicity to BV-2 cells (Figure 1A(Fig. 1)). In NO assay as shown in Figure 1B, BV-2 microglial cells treated with LPS (1 µg/mL), significantly (*p <* 0.001) increased the NO levels when compared with control group (from 2.86 ± 0.86 to 32.64 ± 2.06 µM) which was attenuated with CSE at indicated concentrations (25, 50 and 100 µg/mL) significantly (*p <* 0.05 ~ *p <* 0.001) in a dose-dependent fashion (24.31 ± 2.98, 18.32 ± 1.76, and 10.98 ± 1.12 µM at 25, 50 and 100 µg/mL, respectively). The lowest dose of CSE tested (10 µg/mL) did not affect the NO release in LPS-induced BV-2 microglial cells. Therefore, the non-toxic and effective concentrations of CSE (25, 50 and 100 µg/mL) were used in further experiments.

### Effect of CSE on iNOS and COX-2 expression in LPS-induced BV-2 cells

**Figure 2:**
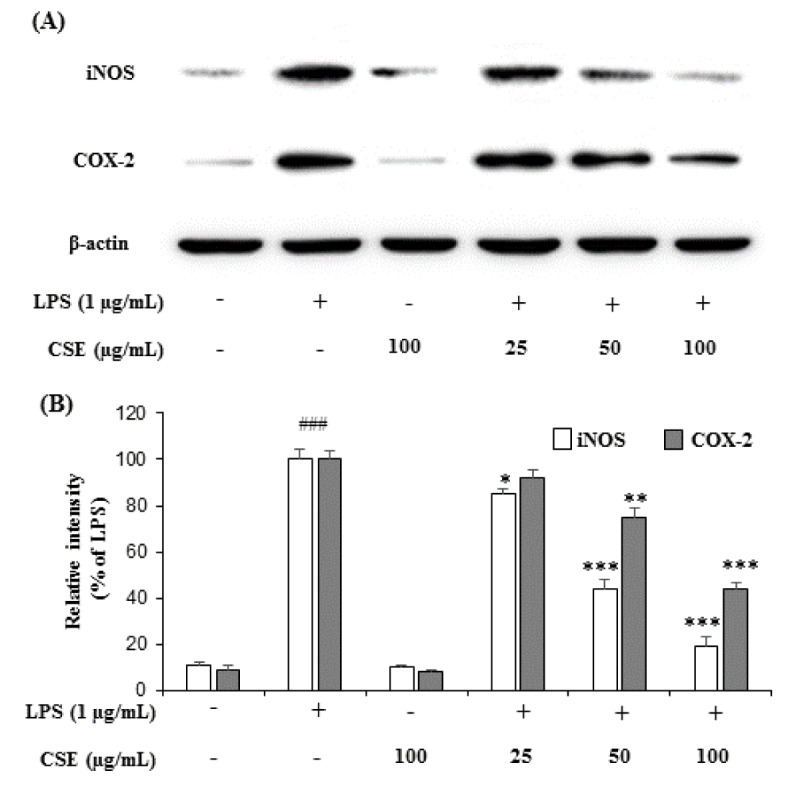
Effect of CSE on iNOS and COX-2 protein expression levels in LPS-stimulated BV-2 cells. The expression levels of iNOS and COX-2 production in the LPS-stimulated BV-2 cells by CSE at indicated concentrations (25, 50 and 100 μg/mL) was evaluated by western blot analyses with the specific antibodies against iNOS and COX-2 (A). Relative intensity levels (percentage of LPS) for three independent experiments (B). The internal control used was β-actin. Values are mean ± S.E.M (n=6). ^###^*p <* 0.001, vs. control group and ^*^p < 0.05, ^**^p < 0.01 and ^***^p < 0.001 vs. LPS treated group. CSE: *Coriandrum sativum *extract

Treatment with LPS (1 µg/mL) markedly increased the expression of iNOS and COX-2 in BV-2 microglial cells when compared with the control group. However, CSE at indicated concentrations (25, 50 and 100 µg/mL) attenuated the increased expression of iNOS and COX-2 in a dose-dependent manner in LPS-induced BV-2 cells (Figure 2A(Fig. 2)). The relative intensities of iNOS were reduced to 85.58 ± 1.14 %, 44.26 ± 4.22 % and 19.84 ± 3.96 %, and COX-2 levels to 92.57 ± 3.16 %, 75.46 ± 3.68 %, 44.42 ± 2.90 % at 25, 50 and 100 µg/mL, respectively compared with LPS-induced groups (Figure 2B(Fig. 2)). 

### Effect of CSE on IL-6 and TNF-α production in LPS-induced BV-2 cells

**Figure 3:**
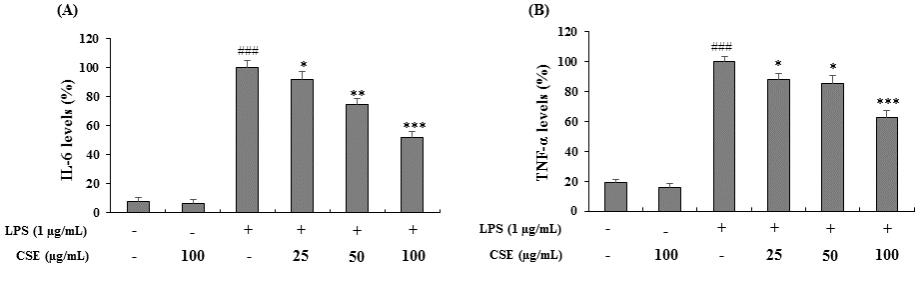
Effect of the CSE on IL-6 and TNF-α levels in LPS-induced BV-2 cells. The amounts of IL-6 and TNF-α in the culture supernatant were measured using ELISA kits. The cells were treated with indicated concentrations of CSE (25, 50 and 100 μg/mL) with or without LPS. Data are expressed as a percentage of activation or inhibition with LPS only-treated cells considered as 100 %. Data are the mean ± S.E.M (n =6). ^###^*p <* 0.001, vs. control group and ^*^p < 0.05, ^**^p < 0.01 and ^***^P < 0.001 vs. LPS treated group. CSE: *Coriandrum sativum *extract

BV-2 cells were stimulated with LPS (1 µg/mL) in the presence or absence of CSE at indicated concentrations (25, 50 and 100 µg/mL) and the levels of IL-6 and TNF-α were measured. As shown in Figure 3A and 3B(Fig. 3), LPS-induced BV-2 cells increased the IL-6 and TNF-α levels significantly (*p <* 0.001) expressed as the percentage of LPS only treated cells. Treatment with CSE at indicated concentrations suppressed the LPS-induced increased levels of IL-6 and TNF-α significantly in a dose-dependent manner (*p <* 0.05, *p <* 0.01 and *p <* 0.001 at 25, 50 and 100 µg/mL, respectively). 

### Effect of CSE on NF-κB levels and IκBα phosphorylation in LPS-induced BV-2 cells 

**Figure 4:**
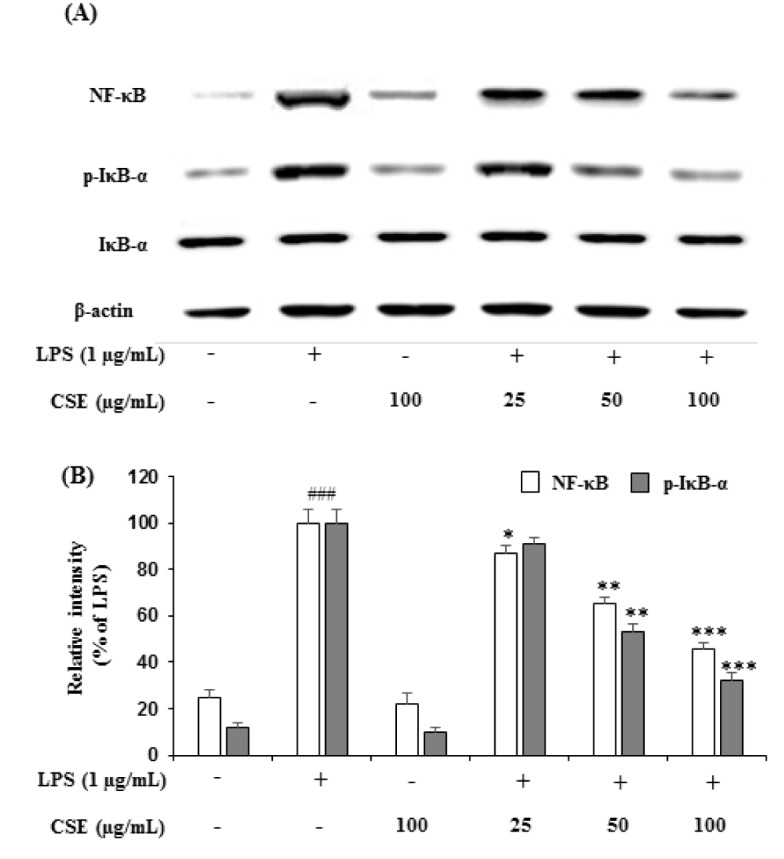
Effect of the CSE on NF-κB and IκB-α expression in LPS-induced BV-2 cells. The expression levels of NF-κB and IκB-α in the LPS-stimulated BV-2 cells by indicated concentrations of the CSE (25, 50 and 100 μg/mL) was analyzed by western blot analyses with the specific antibodies (A). Relative intensity levels (percentage of LPS) for three independent experiments (B). The internal control used was β-actin. Data are the mean ± S.E.M (n =6). ^###^*p *< 0.001, vs. control group and ^*^p < 0.05, ^**^p < 0.01 and ^***^p < 0.001 vs. LPS treated group. CSE: *Coriandrum sativum *extract

To further understand the underlying mechanisms of CSE in inhibiting the LPS-induced iNOS, COX-2 and other proinflammatory cytokines such as TNF-α and IL-6, the LPS-induced NF-κB activation and IκB-α phosphorylation were estimated in BV-2 cells. As shown in Figure 4A(Fig. 4), LPS-induced BV-2 cells markedly increased the expression of NF-κB and IκB-α phosphorylation (p-IκBα). However, CSE treatment inhibited the LPS-induced changes in the expression of NF-κB and suppressed the phosphorylation of IκB-α in a concentration-dependent manner. Relative intensities showed that CSE (25, 50 and 100 µg/mL) attenuated the increased levels of NF-κB to 87.24 ± 3.68 % (*p* < 0.05), 65.48 ± 2.94 % (*p* < 0.01) and 46.28 ± 2.54 % (*p* < 0.001), and p-IκB-α levels to 91.46 ± 2.68 %, 53.24 ± 3.56 % (*p* < 0.01) and 32.12 ± 3.45 %, significantly (*p <* 0.001) in a dose-dependent manner (Figure 4B(Fig. 4)). 

### Effect of CSE on MPTP-induced rotarod performance test in mice

**Figure 5:**
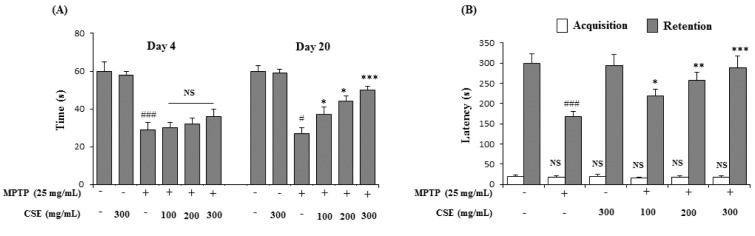
Effect of CSE on the rotarod and passive avoidance task in MPTP-induced mice. Rotarod performance in different experimental groups on day 4 and day 20 was shown (A). For passive avoidance task, the latency times (s) in acquisition (trial 1) was carried on day 20 and retention (Trial 2) was carried 24 h after trial 1 (B). ^#^*p *< 0.05 compared with their respective control groups. NS: Not significant; ^*^p < 0.05, ^**^p < 0.01 and ^***^p < 0.001, compared with MPTP-induced group. Data expressed as mean ± S.E.M (n = 10) using one-way ANOVA followed by Dunnett's test; MPTP: 1-methyl-4-phenyl-1,2,3,6-tetrahydropyridine; CSE: *Coriandrum sativum *extract

In rotarod performance test, the MPTP-induced group significantly (*p <* 0.001) impaired locomotor activity by reduction in their retention times on day 4 (29 ± 2 s) and day 20 (27 ± 3 s) when compared with their respective control groups. Administration of CSE at various doses (100, 200 and 300 mg/kg) did not influence the MPTP-induced locomotor deficits at day 4. However, on day 20, CSE administration (100, 200 and 300 mg/kg) in MPTP-induced groups significantly increased the retention times in a dose-dependent fashion (37 ± 4 s; *p <* 0.05, 44 ± 3 s; *p <* 0.05 and 51 ± 2 s; *p <* 0.001 at 100, 200 and 300 mg/kg, respectively) when compared with MPTP only group (Figure 5A(Fig. 5)).

### Effect of CSE on MPTP-induced passive avoidance test in mice

In passive avoidance task, the latency to enter the dark chamber was not significant in all groups in the absence of the aversive foot-shock stimulus (acquisition trial). In the retention trial, the latency time was increased significantly (300 ± 24 s; *p <* 0.001) when compared to acquisition trial (19 ± 4 s) in control trained group. MPTP administration significantly (*p <* 0.001) reduced the latency time to 167 ± 14 s, compared to the control group. Administration of CSE at indicated doses dose-dependently ameliorated the retention, with a significant improvement in the decreased latency times, compared to the MPTP-induced group (218 ± 8 s; *p <* 0.05, 258 ± 9 s; *p <* 0.01 and 289 ± 8 s; *p <* 0.001, at 100, 200 and 300 mg/kg, respectively). CSE at 300 mg/kg treated dose in MPTP-induced mice group showed the highest effect in reinstating the latency time to near normal levels (Figure 5B(Fig. 5)). 

### Effect of CSE on open field test in MPTP-induced mice

**Figure 6:**
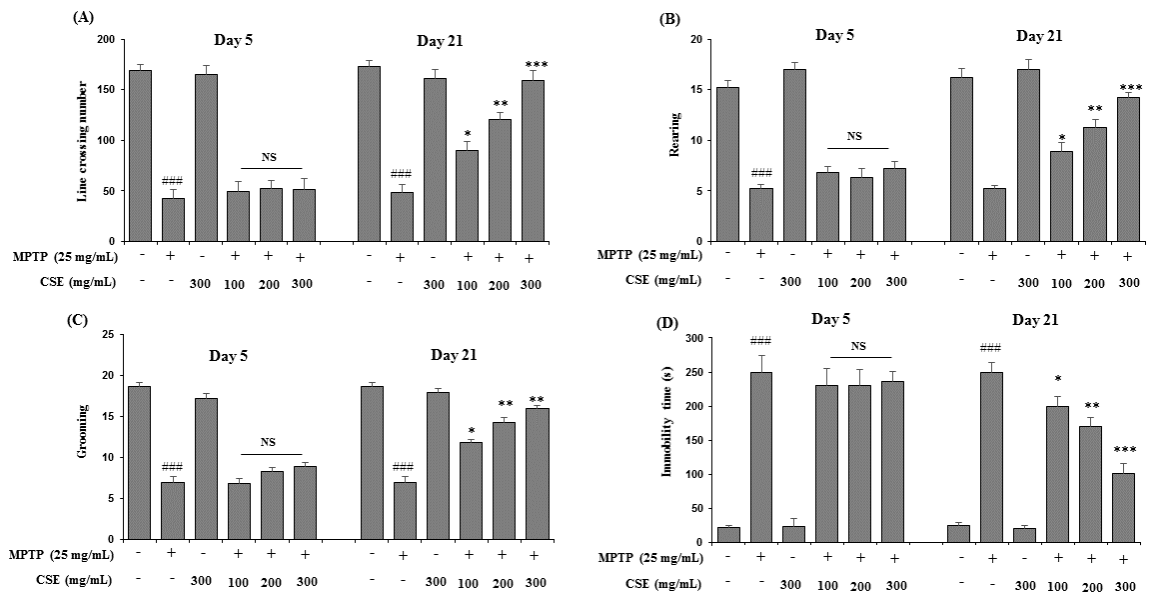
Effect of CSE on the open-field performance in MPTP-induced mice. Cognitive parameters in open field test including the line crossing (A), rearing (B), grooming (C) and immobility time (D) behavior were measured in different experimental groups. ^###^*p <* 0.05 compared with untreated group; NS: Not significant; ^*^p < 0.05, ^**^p < 0.01 and ^***^p < 0.001compared with MPTP-induced group. Data are expressed as mean ± SEM (n = 10) using one-way ANOVA followed by Dunnett's test. MPTP: 1-methyl-4- phenyl-1,2,3,6-tetrahydropyridine; CSE: *Coriandrum sativum *extract.

In open field test, the parameters including the line crossings, rearing, grooming and immobility were observed (Figure 6A-6D(Fig. 6)). MPTP-induced mice showed reduced activities in all the four parameters tested significantly (*p <* 0.001) when compared with control group on day 5 and day 21. However, on day 21, CSE administration (100, 200 and 300 mg/kg) in MPTP-induced groups significantly attenuated the reduced numbers of line crossing, rearing and grooming behavior in mice (*p <* 0.05, *p <* 0.01 and *p <* 0.001 at 100, 200 and 300 mg/kg, respectively). The increased immobility time observed in MPTP-induced group was also ameliorated with CSE administration significantly in a dose-dependent manner (*p <* 0.05 ~ p< 0.001). The highest effect was observed at 300 mg/kg dose (p < 0.001). At day 5, no significant differences were observed in the groups treated with MPTP plus CSE compared with the group induced with MPTP alone. CSE only treated groups also showed no significance in all the parameters tested on day 5 and day 21 when compared with the control groups. 

### Effect of CSE on MPTP-induced changes in antioxidative enzyme levels mouse brain tissue

**Figure 7:**
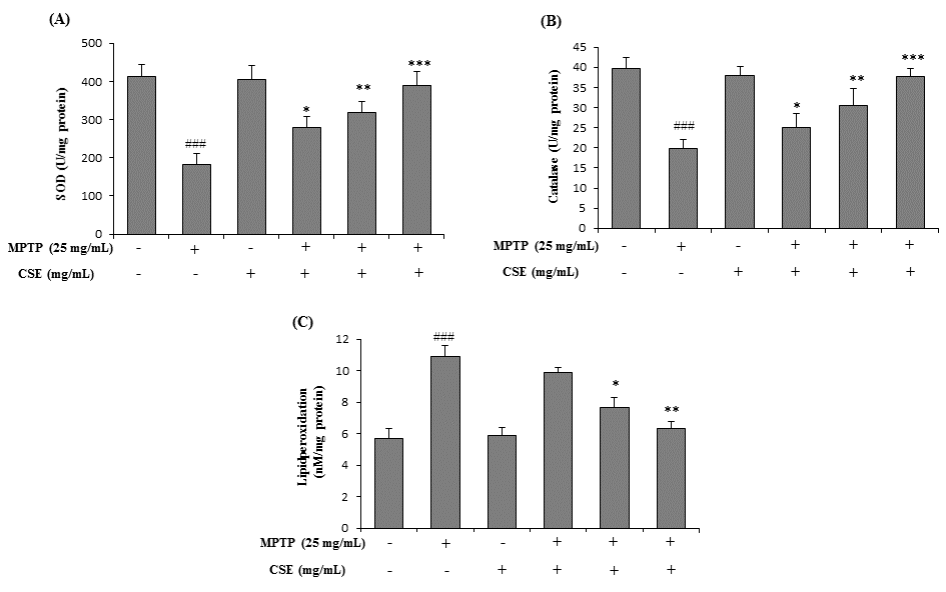
Effect of CSE on the anti-oxidant enzyme levels in MPTP-induced mice. The antioxidant enzymes levels of SOD (A), Catalase (B) and lipid peroxidation (C) in different experimental groups were shown. ^###^*p <* 0.001 compared with untreated group. ^*^p < 0.05 ^**^p < 0.01 and ^***^p < 0.001 compared with MPTP-induced group. Data expressed as mean ± S.E.M. (n=10) using one-way ANOVA followed by Dunnett's tests by Graph Pad Prism v5.01 software. MPTP: 1-Methyl-4-phenyl-1,2,3,6-tetrahydropyridine; SOD: Superoxide dismutase; CSE: *Coriandrum sativum *extract

To evaluate the effect of CSE on antioxidant enzyme status, the levels of SOD, CAT and LPO were estimated in mouse brain tissue. Significant decrease in the activities of SOD and CAT was observed in MPTP-treated group (*p <* 0.001) when compared with their respective control groups (Figure 7A and 7B(Fig. 7)). Further, LPO levels were significantly (*p <* 0.001) increased in MPTP-induced group (Figure 7C(Fig. 7)). Treatment with CSE (100, 200 and 300 mg/kg) dose-dependently attenuated these changes. Although 100 mg/kg dose of CSE did not show significant effect in altering the LPO activity, the effects were dose-dependent. The highest dose with maximum effect in attenuating the MPTP-induced oxidative stress parameters tested was observed at 300 mg/kg dose (p < 0.001).

### HPTLC finger printing profile of CSE

**Figure 8:**
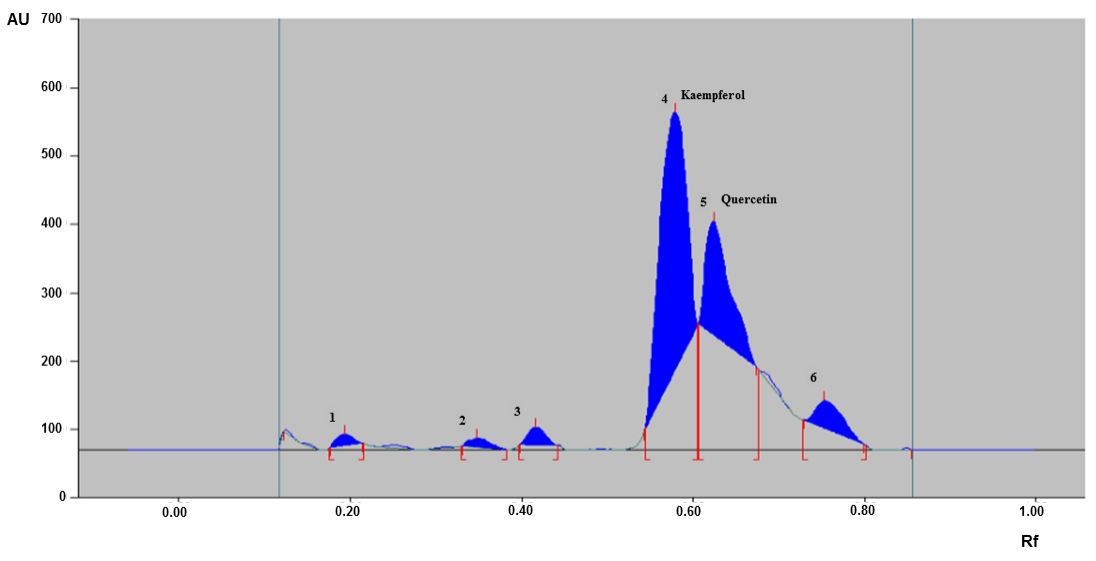
High performance thin layer chromatography (HPTLC) chromatogram of CSE. HPTLC chromatogram of CSE used in the study showing six major peaks of phytoconstituents with peaks 4 and 5 identified as kaempferol and quercetin, respectively. CSE: *Coriandrum sativum *extract; AU: Area under curve; Rf: Retention factor

To develop the finger print of CSE used in the study, HPTLC method was used with the mobile phase of ethyl acetate: methanol: water (8.2:1:0.8) using standard procedure (15 µL) discussed in the materials and methods section and scanned under UV at 254 nm. The HPTLC fingerprinting of CSE used in this study showed six distinctive peaks with varying retention factor (Rf) values in the range of 0.12 to 0.80. Out of the six peaks obtained, peaks 4 and 5 were identified as kaempferol (Rf: 0.53) and quercetin (Rf: 0.61), respectively (Figure 8(Fig. 8)). 

## Discussion

Mounting evidence suggest that microglial activation in the central nervous system as one of the major features of neuroinflammation is seen in PD (Matsumoto et al., 1992[41]; Gao et al., 2002[18]). Neuroinflammation within the selected regions of the brain is led by increased pro-inflammatory cytokine responses and toxic free radicals (McGeer and McGeer, 2004[42]; Block et al., 2007[7]). Regulation of pro-inflammatory mediator and cytokines production in activated microglia might help in mitigating the disease severity (Liu and Hong, 2003[37]; Eikelenboom and van Gool, 2004[14]). Microglial cells are known to be activated in response to various stressors such as LPS in experimental models releasing inflammatory neurotoxic factors (Gao et al., 2002[18]; Orr et al., 2002[48]). Therefore, in this study, LPS-stimulated BV-2 microglia was used as *in vitro *model.

In the present study, LPS-induced increase in the expression of iNOS and excessive release of NO was observed. Further, COX-2 enzyme which plays an important role in the inflammatory neurodegenerative process was also strongly expressed in LPS-induced BV-2 microglia. Increased iNOS and COX-2 expression was known to be pathogenic and enhances the progression of PD (Teismann et al., 2003[62]). However, CSE treatment attenuated the LPS-induced NO production and suppressed the iNOS and COX-2 expression. Activated microglia was known to increase the production of proinflammatory cytokines such as TNF-α and IL-6 (Hofmann et al., 2007[24]). CSE treatment also inhibited LPS-induced production of TNF-α and IL-6 in BV-2 microglial cells in a concentration-dependent manner indicating that CSE might be beneficial in delaying the progression of neuroinflammation.

It is well documented that NF-κB, a transcription factor is involved in regulation of several inflammatory mediators including iNOS and COX-2 (Alderton et al., 2001[2]). Activation of NF-κB in microglial cells triggered by phosphorylation and subsequent degradation of IκB-α plays an important role in regulating the expression of proinflammatory genes including iNOS, COX-2, TNF-α and is majorly involved in the induction of inflammatory responses (Baldwin, 1996[5]). LPS has also been reported to activate NF-κB in microglia (Lee et al., 2004[35]). In this study, CSE inhibited the LPS-induced activation of NF-κB and phosphorylation of IκB-α in a concentration-dependent manner indicating that CSE might regulate the NF-κB signaling pathway.

Previously, the anti-inflammatory properties of *C. sativum* aerial parts and coriander oil were reported in cellular and animal models. *C. sativum* aerial parts at the doses ranging from 100 to 500 mg/kg showed antiinflammatory properties in cotton pellet-induced granuloma, Carrageenan-induced paw edema test, indomethacin- and acetic acid-induced colitis in experimental animals (Jagtap et al., 2004[28]; Zanusso-Junior et al., 2011[73]; Begnami et al., 2018[6]). The anti-inflammatory potential of a lotion containing coriander oil in ultraviolet erythema test was also reported (Reuter et al., 2008[55]). Further, *C. sativum* leaf and stem extracts showed suppressive effects on LPS-induced inflammatory responses in murine RAW 264.7 macrophages at concentration of 150 µg/mL (Wu et al., 2010[70]). In agreement with previous reports that CSE possess antiinflammatory effects, our current study further confirmed that CSE ameliorated neuroinflammatory responses in LPS-induced BV-2 microglial cells at relatively lower concentrations (25-100 µg/mL).

Earlier reports revealed that the neurotoxin MPTP induces microglial activation releasing inflammatory associated factors causing dopaminergic neuronal death and produces marked behavioral impairments in animal models (Block et al., 2007[7]; Kumar et al., 2013[34]). MPTP was well reported to produce Parkinsonian syndrome with notable oxidative stress and behavioral impairments in experimental studies (Tillerson et al., 2002[65]; Watanabe et al., 2008[69]). Therefore, MPTP-induced PD model was used to evaluate the neuroprotective effects of CSE* in vivo*. In the present work, CSE treatment, attenuated the cognitive and behavioral impairments in MPTP-induced mouse model of PD. The doses (100, 200 and 300 mg/kg) selected in this study was based on our earlier work (Koppula and Choi, 2012[33]).

In locomotor behavioral test assessed by rotarod apparatus, decreased retention time on an accelerating rotarod was observed in mice injected with MPTP indicating reduced balance and muscular coordination (Moon et al., 2009[46]). Since the behavioral effects are linked with the degree of neurodegeneration, its assessment is a more powerful endpoint in evaluating neuroprotection. Further, MPTP-induction to mice was known to develop cognitive deficits (Yabuki et al., 2014[71]). In the present study, the cognitive performance was significantly altered by MPTP treatment with poor performance in passive avoidance and open field tasks. However, the performances of mice in both tasks in MPTP-induced mouse significantly improved with CSE treatment. Although no apparent improvement was observed on day 4 (rotarod test) and day 5 (open field test) in behavioral performances, the results of the behavioral tests exhibited a significant improvement on day 20 and 21, respectively suggesting that long-term CSE treatment has a therapeutic effect on MPTP-induced mice.

Oxidative stress is widely considered to be important in the development and progression of PD (Jenner and Olanow, 1996[31]; Zhang et al., 1999[75]). MPTP was known to damage mitochondria, proteins and lipids thereby altering the antioxidant enzyme status in the brain (Perier and Vila, 2012[52]). Therefore, estimating the antioxidant enzyme status in brain tissues might be key parameters in understanding the degree of MPTP-intoxication in animal models of PD. In the present study, the oxidative stress in MPTP-induced parkinsonian mice was measured by determining the activity of SOD, CAT and LPO levels in the mouse brain tissues. CSE extract treatment restored the levels of antioxidant enzymes thereby reducing the oxidative damage caused by MPTP intoxication in mice. The improved cognitive and behavioral functions were well supported by the reduced brain oxidative stress by CSE in MPTP-induced mice.

Finger print analysis by various analytical techniques is a useful approach for the identification of major chemical compounds in various herbs and in quality control of herbal products (Teo et al., 2013[64]; Cortés et al., 2014[10]). Therefore, we performed HPTLC finger print analysis to assess the major phytonutrients present in CSE. Coriander fruits are loaded with nutrients such as high content of antioxidants, flavonoids, polyphenolic compounds, glucosides, volatile oils, dietary fiber, vitamins, copper, potassium, manganese, magnesium, calcium, iron and zinc with moderate amounts of fat and protein (Prachayasittikul et al., 2018[54]). Data showed several sequence of distinctive peaks of CSE and out of which quercetin and kaempferol were identifiable. It is well documented that quercetin and kaempferol possess immense pharmacological benefits including antioxidant, antiinflammatory and neuroprotective effects (Yu et al., 2013[72]; Pany et al., 2014[50]). The active constituents observed in CSE along with other components might act synergistically or in a harmonized manner in delivering potential benefits against microglia-mediated neuroinflammation and exhibited neuroprotective effect in MPTP-induced PD mice.

In conclusion, the present study provides scientific support that CSE attenuates LPS-induced inflammatory responses in BV-2 microglial cells. Further, CSE also ameliorated the MPTP-induced behavioral impairments and oxidative stress parameters in mouse model of PD, indicating its potential in the treatment and management of neuroinflammatory and cognitive behavioral deficits seen in PD. 

## Acknowledgements

This paper was supported by Konkuk University in 2020.

## Conflict of interest

The authors declare no conflicts of interest.
